# The Primary Surgery of Gen. Sherman’s Campaigns

**Published:** 1866-06

**Authors:** E. Andrews, J. M. Woodworth

**Affiliations:** Prof. of Surgery in Chicago Medical College; Late Medical Director of the Army of the Tennessee


ARTICLE XXI.

### THE PRIMARY SURGERY OF GEN. SHERMAN'S CAMPAIGNS.

By E. ANDREWS, M.D., Prof. of Surgery in Chicago Medical College, and J. M. WOODWORTH, M.D., Late Medical Director of the Army of the Tennessee.

A perfect record of the primary surgery of a military campaign is a desideratum seldom obtained. Owing to the hurry of battle and the movements of troops, records on the spot are too often neglected, and any perfect estimate of the value of the various surgical operations rendered impossible. The authors of this article, who were both under the command of Gen. Sherman, viewed with great regret the almost total loss of surgical statistics which occurred in the first half of the war, and resolved on making a strong effort to preserve reliable records of the army in which they served. For this purpose, they made systematic preparations before any similar system was established by the War Department, and succeeded in obtaining a large mass of valuable information which has never reached the Surgeon-General's office. At a later period, the surgical records of the army underwent a reform, and furnished the means of obtaining still larger collections of material.

The subjoined tables contain almost the entire primary surgery of Gen. Sherman's army, during the campaigns in Georgia, leading to the capture of Atlanta, the famous "March to the Sea," the siege of Savannah, and the operations in South and North Carolina to the close of the war. With this is combined a complete record of the battle of Chickasaw Bayou, near Vicksburg, a portion of the other engagements in that region, and a large number of facts, gathered from Shiloh, Corinth, and other Western battles. The death or recovery of each patient is given, so far as obtainable, and if unknown is so stated.

Gen. Sherman's troops were always remarkable for the immense marches which they undertook. They were kept in the lightest possible moving order, and, to a great extent, dispensed with the use of tents, except when stationary. Their history is briefly this:—

After the battle of Shiloh, they moved on Corinth and constituted the extreme right at the capture of that place. They then moved leisurely to Memphis, fortified that place, and occupied the surrounding region, engaging in many small skirmishes, but meeting no large body of the enemy. Thence, they moved by water to Vicksburg, where they engaged in the battles of Chickasaw Bayou, Arkansas Post, siege of Vicksburg, and the other important operations in that region. At the close of this campaign, they returned by water to Memphis, and thence undertook that prodigious march from the City of Memphis almost to Knoxville, and thence back again to Chattanooga. In this region were fought the battles of Mission Ridge, Resaca, Allatoona Pass, Kenesaw Mountain, Jonesboro, etc., etc., resulting in the capture of Atlanta, after a most magnificent campaign. After making proper arrangements, Gen. Sherman sent to the rear all his sick, wounded, and enfeebled men, and, with a force of over 65,000 seasoned and hardy troops, commenced his renowned "March to the Sea." On this march there occurred some sharp fighting, but, owing to the splendid arrangements of Med. Director MOORE, all the wounded were carried through, not a man being left behind. There was an opportunity here to observe the effects of land transportation upon wounded men, and to compare them with the results previously seen on the hospital steamers of the Mississippi River. The results were calculated to overthrow some of the received opinions on this subject. These wounded, though carried in ambulances an immense distance, suffered a much less mortality than the average of the hospital steamers, and even of many of the general hospitals. We infer from this, not that wheel transportation is free from all injurious effects, but that the pure air of an open ambulance, in spite of its shaking, renders it a safer place for a wounded man than the foul air of many general hospitals and hospital steamers, notwithstanding their quietness, their good food, and their soft cots. Where a portion of the wounded of a battle are taken in ambulances to a general hospital at some distance, and another portion are kept in the field, it is uniformly found that those who are treated in the hospital do worst. This is often attributed to the shaking received on the route from the ambulance. The experience of the "March to the Sea" shows, however, that wounded men carried in ambulances from the day of battle to the termination of the case, *do better than in most general hospitals.* The prime necessity of a wounded man is pure air, and compared with this, all questions of diet, beds, and even shelter and repose seem to be secondary. This great truth was difficult to learn, and, to many of us, hard to believe, until it was taught by the stern experience of war, and proved by observations covering many thousand cases.

At the close of the war, marked by the surrender of Johnson's army in North Carolina, Sherman's forces marched to Washington. During this movement, an illustration of the evils of forced marches was displayed on a large scale. The troops were in the highest exhilaration of spirits, at their magnificent conquests and at the prospect of a speedy return home. Acting under this natural impulse, the 15th and 17th Army Corps virtually ran a footrace of several days duration, to see who should reach Richmond first. From 15 to 17 miles is a good day's march for infantry, and very few generals can get large bodies of men over as great an average distance as that, when moving day after day for a considerable time. On the 1st of May, these two corps marched 20 miles; on the 2d, 25 miles; on the 3d, 13 miles; on the 4th, 24½ miles; on the 5th, 18 miles; and on the 6th, 26 miles, when they halted near Petersburg. As the result of this enormous footrace of six days duration, 1630 men were found disabled from lameness and exhaustion, and had to be sent to Washington by steamers.

### THE NUMBER OF THE WOUNDED.

The total number of wounded on our records, is 6122. Of these, 1042 are simply entered as wounded, with no additional information respecting them.

CAUSES OF WOUNDS.

The cause of the wounds is recorded in 4480 cases, as shown in the following table:—

| | |
|---|---|
| Bullets, (nearly always conical,) | 3980 |
| Shells, | 384 |
| Buckshot, | 25 |
| Grape and Canister, | 24 |
| Torpedoes, | 14 |
| Pistol Shots, | 12 |
| Solid Shot, | 11 |
| Accidental Explosions, | 8 |
| Bayonets, | 7 |
| Splinters, | 6 |
| Stones, | 3 |
| Gun Caps, | 3 |
| Strippings of Cannon Shot, | 3 |
| Sabres, | 0 |
| Total, | 4480 |

It will be seen at a glance, that the musket is manifold the most destructive agent used in war, causing seven-eighths of all the wounds. This, however, is owing purely to the numerical preponderance of the infantry. If we examine the table, it will be seen that 431 wounds were caused by artillery, which is about 10 per cent of the whole number. Now it is not probable that the enemy's artillery exceeded 7 per cent of his entire force, which shows that, in proportion to the men engaged, artillery is more destructive than infantry and cavalry. It is seen, also, that almost the whole effect of artillery is produced by shells, the wounds of which were 384 in number, while the solid shot, grape, canister, splinters, stones, and strippings of shot, altogether, only injured 47 men.

The buckshot are mostly from shotguns, in the earlier half of the war, when that weapon was extensively carried by the rebels. The remainder are from the old-fashioned round musket ball cartridge, also much used by the enemy. The uselessness of the buskshot is shown by the few wounds produced by them. The gun cap wounds were in the eye, and were caused by the bursting of the percussion cap on the nipple of the gun.

Only 7 bayonet wounds are recorded, and a part of these were accidental. It will be remembered that though bayonet charges are often made, men seldom cross these weapons in close fight. One party or the other gives way before the collision occurs.

The 14 torpedo wounds were all received in the storming of Fort McAllister, near Savannah, from torpedoes planted in the earth to assist in the defence. The pistol wounds were almost all received in the same assault, in consequence, doubtless, of the rebel officers using their pistols to increase the amount of fire from the walls.

Not a single sabre wound is recorded, though these weapons were carried by all the cavalry and many of the artillery. The rebels, at the beginning of the war, had high expectations for their cavalry, owing to the great prevalence of habits of saddle travel in that region; but this, like many other mistakes, arose from not considering all their circumstances. The fact is, the nature of the country is such, that extensive cavalry operations, in the European sense of the term, are impossible. Almost every battle was either in the forest, or among such crags, ravines, or marshes that an opportunity for a regular charge by mounted men could scarcely ever be found. A body of highly-trained European cavalry placed in our Southern States would have been the most helpless piece of lumber that ever encumbered an army—incapable of defence, and useless for attack. Both sides, therefore armed their mounted forces with carbines, and sabres rusted idly in their scabbards.

DISTRIBUTION OF THE INJURIES.

The following table shows the nature and position of injuries, with their results, so far as traced:—

| Wound. | Recov'd. | Died. | Result Unk'n. |
|---|---|---|---|
| Flesh wounds of head,------------------ | 146 | 10 | 69 |
| Non-penetrating flesh wounds of trunk, | 214 | 7 | 87 |
| Penetrating wounds of thorax, -------- | 26 | 127 | 76 |
| " " abdomen,------ | 7 | 109 | 31 |

| | | | |
|---|---|---|---|
| “ “ pelvic viscera,- | | 4 | |
| Flesh wounds of superior extremity,-- | 221 | 2 | 78 |
| “ “ inferior “ -- | 425 | 9 | 81 |
| Wounds of large arteries,-------------- | 5 | 3 | 2 |
| Contusions,-------------------------- | 102 | 4 | 34 |
| Concussions,------------------------- | 6 | 2 | 2 |
| Fractures of cranium, not penetrating the brain,---------------------- | 15 | 9 | 7 |
| Penetrating fractures of cranium,----- | | 54 | 7 |
| Comp. fractures of facial bones,------ | 45 | 8 | 15 |
| Fractures of spine,------------------- | 2 | 21 | 3 |
| Compound fracture of scapula,-------- | 10 | | 5 |
| “ “ clavicle,-------- | 1 | 3 | |
| “ “ shoulder-joint,-- | 63 | 20 | 17 |
| “ “ humerus,------ | 101 | 24 | 55 |
| “ “ elbow-joint, ---- | 29 | 7 | 17 |
| “ “ forearm,------- | 88 | 8 | 50 |
| “ “ wrist,---------- | 13 | | 8 |
| “ “ hand & fingers,- | 124 | 2 | |
| “ “ pelvis,--------- | 7 | 10 | 11 |
| “ “ hip-joint, ------- | | 9 | 1 |
| “ “ femur,--------- | 78 | 125 | 57 |
| “ “ knee-joint, ----- | 24 | 45 | 41 |
| “ “ leg,------------ | 91 | 45 | 73 |
| “ “ ankle-joint, ---- | 14 | 1 | 8 |
| “ “ foot,----------- | 53 | 3 | 23 |
| Wounds not described, mostly flesh-wounds which usually recovered,- | | | 2683 |
| | 1910 | 671 | 3541 |

In looking over the above list, it will be seen that it is impossible to determine the absolute ratio of mortality, owing to the great number of cases in which the results are unascertained. As the greater part of the deaths occur within 20 days of the injury, it is to be presumed that the deaths were much fewer among the cases which went to the rear and were lost sight of, than among those of which the result was ascertained. As a test of relative mortality, however, the table is valuable.

### FLESH WOUNDS.

Simple flesh wounds, not involving large vessels, nerves, nor important cavities, produce very little danger. Of 1034 flesh wounds whose results were ascertained, only 28 died, or a little over 2 per cent.

### PENETRATING WOUNDS OF THE CHEST.

The danger of this class of injuries is in striking contrast to the safety of the preceding. Of 153 cases, whose results were ascertained, 127 died, and only 26 recovered, being a mortality of about 83 per cent. As there were 76 cases whose termination was not ascertained, it is probable that a larger portion of them recovered, which would reduce the rate somewhat. The Surgeon-General, from a list of 1272 cases, reports a mortality of 73 per cent.

### PENETRATING WOUNDS OF THE ABDOMEN.

These are still more fatal than those of the chest. Out of 116 cases 109 died, making a mortality of 94 per cent, while only 31 cases remained in doubt. The Surg.-Gen.'s statistics differ widely from these. In a list of 543 cases he reports a mortality of only 74 per cent, which is only a trifle greater than that of the chest wounds. As this is contrary to all our personal observation, we are compelled to believe that the Surg.-Gen.'s statistics must be vitiated by cases of mere flesh wounds of the parietes, falsely reported by careless and hurried surgeons as penetrating wounds. This is the more likely, as the diagnosis is not always easy, and conjectures are often put into official reports instead of knowledge, in the hurry of field duty. Certainly there must be a great error somewhere in figures which make penetrating wounds of the abdomen scarcely more dangerous than those of the chest.

We found the proportion of deaths so great that we seriously contemplated the heroic experiment of opening the abdominal cavity, washing it out, and stitching the wounded intestine to the external orifice, as a desperate resort for a desperate injury; but not finding any authority to justify us, we did not finally venture on it. We deem the suggestion, however, worthy of serious consideration. Wounds of the large intestine appear, for some reason, to be more hopeful than those of the small bowel.

### WOUNDS OF THE PELVIC VISCERA.

These were few in number and uniformly fatal, in our recorded cases. We learned, however, of cases that recovered in other departments.

### FRACTURES OF THE CRANIUM.

These fractures should be considered in two classes, viz.:—the penetrating, and the non-penetrating. Those in which a bullet, or even a buckshot, penetrates the brain are the worst of all wounds. Out of 61 cases on our list, 54 are known to have died, not a single one was ascertained to have recovered, and only 7 remained in doubt. The cases, however, in which the bullet only fractured the bone and lodged or glanced off without penetrating the brain were widely different in their results. Out of 24 cases only 9 died. The Surg.-Gen.'s reports mix both these classes together, so that it is impossible to compare his results with our observations. In two of the non-penetrating fractures, epilepsy is ascertained to have ensued.

### FRACTURES OF THE SPINE.

Out of 26 cases 21 died, only 2 recovered, and 3 were unascertained. Entirely similar to this is the official record of the Surg.-Gen., who has a list of 187 cases, out of which 180 died.

### FRACTURES OF THE SCAPULA AND CLAVICLE.

Fractures of the scapula gave us no ascertained case of death, but out of 4 fractures of the clavicle 3 died; but the fatal result in these cases was owing to penetration of the lung, and other complications.

### FRACTURES OF THE SUPERIOR EXTREMITY.

These are not very dangerous, and generally do well under conservative treatment. The table shows that the danger diminishes as the location of the injury recedes from the shoulder.

### FRACTURES OF THE HIP-JOINT.

Of 9 ascertained cases, not one recovered. The statistics at Washington show similar results. Of 82 cases, only 2 survived.

### FRACTURES OF THE FEMUR.

These are a most dangerous class of injuries. The brittle, ivory like tissue of the shaft of the femur is often comminuted by the force of a bullet into a hundred fragments, which are spread out into the muscular tissue of the thigh with all the force of an explosion. The shock, therefore, is tremendous, and a large portion of the patients sink without reaction. Great efforts have been made to improve this result, by new modes of treatment, but still the fact remains that the majority of gunshot fractures of the femur are fatal. Our table shows 78 recoveries and 125 deaths. The report of the Surg.-Gen., in like manner, shows 462 recoveries and 689 deaths, which gives nearly the same ratio.

### FRACTURES OF THE KNEE-JOINT.

These also are fearful injuries. Out of 69 ascertained cases, we lost 45, which is a mortality of 65 per cent. The tables of the Surg.-Gen. are much less favorable. They show 598 deaths out of 770 cases, which is a mortality of nearly 78 per cent. This result is mainly attributable to the fact that almost all our cases were treated by primary amputation, while many of the Surg.-Gen.'s cases were treated conservatively, which in this injury is synonymous with destructively.

### AMPUTATIONS.

The following table shows the cases of amputation in which the results were ascertained:—

| | Recovered. | Died. | Per cent of Deaths. |
|---|---|---|---|
| Primary amputation at Shoulder-joint,--- | 11 | 2 | 15 |
| Secondary " " --- | 2 | 1 | 33 |
| Primary " Arm,------------ | 55 | 5 | 8 |
| Secondary " " ------------ | 4 | 1 | 20 |
| Primary " Forearm,-------- | 21 | 2 | 9 |
| Secondary " " -------- | | 1 | |
| Amputation at Hand,-------------------- | 4 | 1 | 20 |
| " Fingers,----------------- | 60 | 1 | 1½ |
| Primary amputation, Upper 3d of Femur, | 5 | 13 | 72 |
| Secondary " " " | | 2 | |
| Primary " Middle 3d " | 11 | 14 | 56 |

| | | | | | |
|---|---|---|---|---|---|
| Secondary | " | Middle 3d of Femur, | 2 | 2 | 50 |
| Primary | " | Lower " " | 37 | 24 | 40 |
| Secondary | " | " " " | 24 | 12 | 33 |
| Primary | " | Leg, | 60 | 24 | 29 |
| Secondary | " | " | 7 | 4 | 36 |
| Amputation at Ankle-joint, | | | 3 | 1 | 25 |
| Pirrogoff's amputation, | | | 2 | 1 | 33 |
| Syme's | " | | | 1 | |
| Chopart's | " | | 2 | | |
| Amp. at other parts of Foot and Toes, | | | 18 | 1 | 5 |

The following figures, extracted from Circular No. 6, of the War Department, will serve as a basis of comparison with the above:—

| | Recovered. | Died. | Per cent of Deaths. |
|---|---|---|---|
| Amp. of Fingers and parts of Hand, | 1778 | 29 | 1.60 |
| " at Elbow, | 19 | | |
| " Forearm, | 500 | 99 | 16.52 |
| " Arm, | 1535 | 414 | 21.24 |
| " Shoulder-joint, | 144 | 93 | 39.24 |
| " Toes, | 784 | 6 | .75 |
| Partial amp. of Foot, | 108 | 11 | 9.24 |
| Amp. at Ankle-joint, | 58 | 9 | 13.43 |
| " Leg, | 1737 | 611 | 26.02 |
| " Knee-joint, | 52 | 64 | 55.17 |
| " Thigh, | 568 | 1029 | 64.43 |
| " Hip-joint, | 3 | 18 | 85.71 |

The following list of amputations is compiled from the records of Surgeon-General CHISHOLM, of the Confederate Army:

| Place of Amputation. | | | Recovered. | Died. | Per cent of Mortality. |
|---|---|---|---|---|---|
| Primary | amputation | Shoulder-joint, | 54 | 25 | 31 |
| Secondary | " | " | 8 | 20 | 71 |
| Primary | " | Arm, | 252 | 42 | 14 |
| Secondary | " | " | 87 | 53 | 37 |
| Primary | " | Elbow-joint, | 3 | 1 | 25 |
| Secondary | " | " | 23 | 6 | 26 |
| Primary | " | Forearm, | 61 | 8 | 12 |
| Secondary | " | " | 35 | 10 | 22 |
| Primary | " | Hip-joint, | 1 | 2 | |
| Secondary | " | | 1 | 1 | |
| Primary | " | Thigh, | 213 | 132 | 38 |
| Secondary | " | " | 43 | 119 | 73 |

| | | | | | |
|---|---|---|---|---|---|
| Primary amputation | Leg, | | 219 | 95 | 30 |
| Secondary " | " | | 76 | 74 | 49 |
| Primary " | Chopart's, | | 13 | 3 | |
| Secondary " | " | | 7 | 1 | |
| Primary " | Syme's, | | 2 | | |
| Secondary " | " | | 4 | | |
| Primary " | Pirrogoff's, | | 2 | 2 | |

AMPUTATIONS AT THE SHOULDER-JOINT.

For this operation we find the following rates of mortality:—

| | | | |
|---|---|---|---|
| In Sherman's Army, prim. and sec., | | 23 | per cent. |
| U.S. Surg.-Gen.'s tables, | " | 39 | " |
| Confederate Army, primary, | | 31 | " |
| British wars with Napoleon " | (Guthrie) | 28 | " |

AMPUTATIONS OF THE ARM.

| | | | |
|---|---|---|---|
| Mortality in | Sherman's Army, prim. and sec. | 10 | per cent. |
| " | Surgeon-General's Tables " | 21 | " " |
| " | Confederate Army, primary | 21 | " " |
| " | European Armies, (Guthrie) | 23 | " " |

We saw no cases of amputation at the elbow.

AMPUTATIONS OF THE FOREARM.

| | | | |
|---|---|---|---|
| Mortality in | Sherman's Army, prim. and sec. | 12 | per cent. |
| " | Surgeon-General's tables " | 17 | " " |
| " | Confederate Army, primary | 19 | " " |

AMPUTATIONS AT THE HIP-JOINT.

This operation was not resorted to in any instance, consequently we can add nothing to the general stock of knowledge on that point. The Surgeon-General reports 21 cases, 18 of which died.

AMPUTATIONS OF THE THIGH.

| | | | Mortality. |
|---|---|---|---|
| Primary Amp. in | Sherman's Army | 49 | per cent. |
| Secondary " | " " | 61 | " " |
| Primary " | Confederate " | 38 | " " |
| Secondary " | " " | 73 | " " |

The Surgeon-General does not distinguish between primary and secondary cases, and, therefore, his statement is not entered here for comparison.

The death-rate varied very much, according to the distance from the body, as the following statement shows:—

| | | | | | | |
|---|---|---|---|---|---|---|
| Mortality of Prim. Amp. in | Upper 3d | of Thigh | ----72 | per cent. | | |
| " " " | Middle " | " | ----56 | " | " | |
| " " " | Lower " | " | ----40 | " | " | |

AMPUTATIONS AT THE ANKLE-JOINT.

These are few in number, but, so for as they go, confirm the superior safety of SYME'S operation, as compared with PIRROGOFF'S. Adding our own figures to those of the rebel army, we find the following result:—

| | Recovered. | Died. |
|---|---|---|
| Syme's Amputation | 6 | 1 |
| Pirrogoff's " | 4 | 3 |

PIRROGOFF, himself, is said to have abandoned his operation, on account of the frequent necrosis of the stump of the os calcis. Certainly, the military record is against it, and yet we have found it in civil practice a very useful and comparatively safe operation.

It will be noticed above, that there is, apparently, a much less mortality in our statistics of amputation than in those of our Surgeon-General, or those of the Confederate Army. This arises, partly, from the fact that our cases are nearly all primary, our observations being mostly confined to field service, while the records of the Surgeon-Generals of both armies contain an excess of secondary operations, from the fact that the general hospital cases were all faithfully reported to them, but the field cases were not so fully recorded. Their tabular results are, therefore, affected by the greater mortality which always belongs to secondary operations. The U.S. Surgeon-General has not stated separately the primary and secondary cases, so that we are unable to compare Sherman's army in this matter with the rest of the Union forces. The Confederate statistics, however, are distinguished into primary and secondary, and still show a greater mortality than Sherman's in most of the amputations.

RESECTIONS.

The following table gives a view of all the resections whose results were determined:—

*Resections in Sherman's Army.*

| Operation. | Recovered. | Died. | Per cent of Deaths. |
|---|---|---|---|
| Prim. Resection of Shoulder-joint | 18 | 8 | 31 |
| Sec. " " | 23 | 5 | 18 |
| Prim. " in continuity of the Humerus | 7 | 1 | 12 |
| Sec. " " " " | 5 | | |
| Prim. " of Elbow | 6 | 2 | 25 |
| Sec. " " | 1 | 1 | |
| Prim. " in continuity of Forearm | 19 | 1 | 5 |
| " " " Femur | | 4 | 100 |
| Sec. " " " | 4 | 4 | 50 |
| Prim. " of Knee | 1 | 1 | |
| Sec. " " | | 1 | |
| Resections in continuity of Tibia | 5 | | |
| " " Fibula | 3 | | |
| " " Clavicle | 1 | 4 | 80 |

*Resections reported by the U.S. Surgeon-General.*

| Operation. | Recovered. | Died. | Per cent of Deaths. |
|---|---|---|---|
| Prim. Resection of Shoulder-joint | 160 | 50 | 23 |
| Sec. " " | 183 | 115 | 39 |
| Resection of continuity of the Humerus | 133 | 42 | 24 |
| " the Elbow | 224 | 62 | 22 |
| " in the continuity of Forearm | 217 | 32 | 13 |
| " of the Hip-joint | 4 | 26 | 87 |
| " in the continuity of the Femur | 6 | 32 | 84 |
| " of the Knee | 2 | 9 | 84 |
| " in the continuity of the Tibia | 48 | 11 | 19 |
| " " " " Fibula | 60 | 15 | 20 |

*Resections in the Confederate Army.*

| Operation. | Recovered. | Died. | Per cent of Deaths. |
|---|---|---|---|
| Prim. Shoulder-joint | 28 | 13 | 27 |
| Sec. " | 19 | 7 | 21 |
| Prim. Elbow-joint | 22 | 3 | 12 |
| Sec. " | 23 | 6 | 24 |
| Hip-joint | 1 | 1 | |
| Knee-joint | 2 | 2 | |

From an examination of these tables, it will be seen that resections do not follow the law of amputations in respect to primary and secondary operations. In Sherman's army, primary resections were generally more fatal than secondary, though the reports from other parts of the army do not fully conform to the same rule.

From comparison of the various tables, it will be seen that resection of the shoulder is preferable, on the score of safety, to amputation, and, accordingly, it was generally given the preference by surgeons, wherever the choice presented itself. In addition to the greater safety of the operation, there is the important consideration of the usefulness of the limb, which, in spite of the loss of bone, is very valuable. The same remarks are applicable to resections of the elbow and wrist. In short, the military surgeon may, in respect to resections of the superior extremity, guide himself by the rules of civil surgery.

The writers of this article had no experience with military resections of the hip-joint, as we deemed that the patient's chance was better without the operation than with it. The Surgeon-General's tables give it a mortality of 87 per cent.

For similar reasons, we abstained from resections of the knee-joint. The impossibility of transporting such patients in an ambulance is obvious at a glance. Wounds of the knee-joint were, therefore, generally treated by amputation at the lower 3d of the thigh. Of three such resections which fell under our notice, two died. The Washington statistics give a mortality of 84 per cent to resection of the knee, while the primary amputation in the lower 3d of the thigh only gave, in Sherman's army, a mortality of 40 per cent.

### RESECTION IN THE CONTINUITY OF BONES.

This class of resections is seen in the above tables to be considerably more fatal than amputations of the same parts, a fact which is well proved, but difficult to explain. Primary resections in the continuity of the humerus had, among us, a mortality of 12 per cent, and amputations of only 8 per cent. In attempting the same resection on the shaft of the femur, 66 per cent died, which is 10 per cent worse than our experience in primary amputations in the middle of the thigh. Still, in the superior extremity, the use of the hand is so important that the operation may be risked, even on the humerus, in favorable cases, and in the inferior extremities, it is quite proper on the fibula and metatarsals. In the femur, the tibia, and the clavicle it is very fatal, and should not be performed, except in secondary cases accompanied by necrosis.

### CONSERVATIVE SURGERY.

The question whether to operate or trust to nature is often a difficult one, which can only be decided by knowing what the risk is in cases not operated upon. The following tables throw a good deal of light upon this subject:—

GUNSHOT FRACTURES TREATED WITHOUT OPERATION.

| Fracture. | Recov'd. | Died. | Pr ct. Mort. Sh'rm'n's Ar. | Pr ct. Mort. Sur.-Gen. Rep. | Pr ct. M. Reb. Ar. |
|---|---|---|---|---|---|
| Shoulder-joint | 35 | 7 | 17 | | 35 |
| Humerus | 33 | 7 | 17 | | |
| Elbow-joint | 9 | 3 | 25 | | 11 |
| Forearm | 43 | 1 | 2 | | |
| Wrist-joint | 5 | | | | 7 |
| Hip-joint | 4 | 10 | 71 | 100 | 100 |
| Femur | 64 | 68 | 51 | 63 | |
| Knee-joint | 25 | 42 | 63 | 84 | 51 |
| Leg | 33 | 17 | 34 | | |
| Ankle-joint | 9 | 1 | 10 | | 20 |
| Foot | 21 | 1 | 5 | | |
| Cranium {penetrat'g & non-pen} | 5 | 13 | 78 | | |
| Face | 15 | 1 | 6 | | |

Table of Wounded Joints, treated in the Confederate Army without amputation (from Surg.-Gen. CHISHOLM'S Report):—

| Joint Wounded. | Recovered. | Died. | Joint Anchylosed. | Pr ct. of Deaths. |
|---|---|---|---|---|
| Shoulder | 11 | 6 | 3 | 35 |
| Elbow | 50 | 5 | 22 | 11 |
| Wrist | 26 | 2 | 7 | 7 |
| Hip | | 8 | | 100 |
| Knee | 50 | 53 | 21 | 51 |
| Ankle | 23 | 6 | 1 | 20 |

There is something inexplicable in the Confederate statistics, as given in this table. That out of 103 wounds of the knee-joint, only 21 should become anchylosed, and only 53 died under conservative treatment, is, certainly, contrary to the experience of the rest of the world, and suggests the possibility either of typographical error, or else that some cases of wounds about the knee are included which did not penetrate the cavity of the joint.

The following conclusions are, on the whole, warranted by the figures:—

Conservative treatment of wounds of the shoulder-joint appears, at first glance, to be more successful than either resection or amputation. It is to be remembered, however, that the milder cases were saved and the worse ones operated upon, so that the comparison cannot be pressed. The impression made upon us by our experience, is this:—Cases where the joint is simply opened, or slightly fractured, should be treated without operation. Where the joint is much comminuted, resection should be preferred, and where the comminution is accompanied with destruction of the great axillary nerves and vessels, amputation is imperative.

In the elbow-joint, the mortality of the conservative treatment with us was 25 per cent, that of primary resections, 25 per cent, that of primary and secondary resections together, 33 per cent, and of primary amputation above the elbow, only 8 per cent, showing that amputation is the safest, resection the next, and conservative the most destructive treatment of wounds of the elbow-joint. Our figures for primary amputations above the elbow are more favorable than the experience of the rest of the world bears out. The mortality for primary and secondary amputations of the arm together in the U.S. Surgeon General's report, is 21 per cent, but even this is better than treatment without operation. On the whole, we prefer resection in all gunshot fractures of the elbow-joint in which the principal nerves are intact, but in case of destruction of these, amputation is the best. Conservative treatment will do in very slight fractures.

Compound fractures of the hip-joint were, in our experience, treated conservatively, with a mortality of 71 per cent. The U.S. Surgeon-General, however, as well as the Rebel statistics, gives a mortality of 100 per cent for such treatment. These fractures are not always easily distinguished from extra capsular fractures of the neck of the femur, and it is possible that our results are made too flattering, by the inclusion of some of the latter in the list. The subject is one which needs further investigation.

Fracture of the femur between the articular extremities is a very fatal accident. A large portion of the patients die of shock during the first three days, under any treatment which may be adopted. In comparing conservative treatment with amputation, it must be remembered that fracture of the shaft of a femur, if amputated, requires the operation at the middle or upper third; hence, amputations at the lower third are excluded from the comparison.

Our results are, as follows:—

Mortality of cases treated without operation,--- 51 per cent.
" " " by resection in continuity of the shaft,------------------------ 66 "

Mortality of amputa. at Middle and Upper 3d, 63 "
showing an advantage of 10 or 15 per cent of treatment by splints and extension over operative procedures. We, therefore, were led to discourage amputation for fractures of the femur, except when necessitated by destruction of the nerves and vessels.

Great interest has been excited by the question whether amputation should be performed for penetrating wounds of the knee-joint. Our figures give for primary amputations just above the knee a mortality of 40 per cent; and for treatment of perforated knees, without operation, a death-rate of 63 per cent; giving a balance of 23 per cent in favor of primary amputation.

The Surg.-General recommends primary amputation at the knee-joint, and gives its statistical mortality in our army as 35 per cent. This operation was not practiced under our observation, but the evidence in its favor has become so strong that in future wars it will probably be preferred in most cases of perforated knee-joints.

Treatment of fractures of the leg, without operation, give about the same risk as amputation below the knee. This method, therefore, should be adopted wherever there is hope of preserving a useful limb.

TREPHINING.

Only six cases of trephining were traced to their results. Of these, three were for wounds penetrating the brain, and all died. The other three were for depressed fractures not penetrating the brain, and all recovered. The statements of the Surgeon-General absurdly class the penetrating and non-penetrating fractures together, which renders it impossible to collate his figures with ours. The opinion which we have arrived at is, that non-penetrating gunshot fractures of the cranium require trephining in military as well as in civil practice, when the fragments are depressed, but that cases in which the bullet has passed into the brain are not benefitted by the operation, though as they all died alike, we can scarcely say that any harm was done. In penetrating fractures, the chief mischief is too deep to be helped by the trephine.

ANÆSTHETICS.

The anæsthetic used was almost exclusively chloroform. This agent was administered in about 3000 cases, and produced two deaths. The first death was that of Private J. W. Whitlock, Co. H, 111th Ill. Inf., wounded in the elbow-joint. On *post mortem* examination, the pericardium was found adherent and the lungs consolidated, showing prior disease of both organs.

The second case showed nothing on *post mortem* examination to account for the fatal result.

We have endeavored in these pages, to give a condensed and truthful statement of the materials in our hands, in order that impressions and facts, now fresh and valuable, may not be lost to the world. In conclusion, we wish to put on record an opinion impressed upon us by very large and varied observation, but which it is impossible to put in a tabular form, and it is this:—A very great error has been committed in the anxiety of Medical Directors to gather the wounded into large general hospitals, where cots, white sheets and pillow-cases, and luxurious diet shall contribute to the comfort of the soldier, and where the general air of compactness and good order, and the fine appearance of the buildings shall favorably impress the public. It often happens that those very hospitals, which by their show of cots, sheets, clean floors, and well-cooked meals, extort praise from visitors, newspaper reporters, and even Medical Inspectors, are hothouses of death to the inmates, in consequence of the impossibility of perfect ventilation in the interior. Our experience shows, that in many of these fine hospitals soldiers die in large numbers, who would have recovered had their wounds been treated under a tent or a tree, on their blankets, by their regimental surgeons.

The very first necessity of a wounded soldier is absolutely pure air, such as cannot be had in most of the buildings selected for general hospitals, but when this requirement is fully met, it is astonishing what vigor the system often shows in repairing its injuries. The next great improvement needed in Military Surgery is a system of general hospitals which shall furnish air as pure as that of the open fields. When such a system shall be devised and put in operation, war will be stripped of a large percentage of the mortality at present following in its train.

Note.—The Confederate statistics given are derived from the Richmond *Medical Journal* and from a work issued by the Confederate Surgeon-General, for the instruction of his medical officers.

